# Editorial: Dendritic Cell-Based Immunotherapy in Solid and Haematologic Tumors

**DOI:** 10.3389/fimmu.2020.00507

**Published:** 2020-03-31

**Authors:** Riccardo Dolcetti, Alejandro López-Soto, Jessica Dal Col

**Affiliations:** ^1^The University of Queensland Diamantina Institute, Translational Research Institute, Brisbane, QLD, Australia; ^2^Departamento de Biología Funcional, Inmunología, Universidad de Oviedo, Oviedo, Spain; ^3^Department of Medicine, Surgery and Dentistry “Scuola Medica Salernitana”, University of Salerno, Baronissi, Italy

**Keywords:** dendritic cell vaccination, dendritic cell tolerization, anticancer immunotherapeutic combination, immune checkpoint inhibitor, immunogenic cell death

The safety and feasibility of dendritic cell (DC)-based immunotherapies in the treatment of solid and hematologic tumors are well-documented. However, available clinical data indicate that DC-based vaccination as monotherapy provides suboptimal and still unsatisfactory clinical benefits despite the induction and/or strengthening of specific anti-tumor immune responses. In addition to their function as antigen presenting cells, DCs govern the nature of the immune contexture of tumors owing to the intricate interplay they establish with other immune cell populations (1). Accordingly, recent studies indicated an important role of DCs in mediating the clinical response to immune checkpoint inhibitor (ICI) therapy (2–5) and adoptive CD8^+^ T cell therapy as well (6). In line with these evidences, an emerging area of intense investigation is the development of new and more effective strategies to strengthen the therapeutic efficacy of immune checkpoint blockade with DC-based vaccination. The potential clinical relevance of this combination is supported by convincing preclinical data and clinical trials demonstrating therapeutic synergism and improved efficacy with a retained safety profile (7, 8). This Research Topic of Frontiers in Immunology focuses on the most recent advances in the field of DC-based immunotherapy, a strategy that has recently regained a strong interest as a possible therapeutic complementation of current immunotherapeutic approaches. In this issue, original research by Kodumudi et al. (Brian J. Czerniecki lab) highlighted that the timing and the schedule of ICI treatment and DC-based vaccination are fundamental to reach improved immune response in a preclinical model of HER2^+^ breast cancer. Indeed, the authors clearly showed a reduction in tumor burden and improved survival benefit only when HER2 peptide-loaded DC vaccination preceded anti-programmed cell death 1 (PD-1) therapy. These results were in line with an increase intratumor infiltration of T lymphocytes whose PD-1 expression was up-regulated by DC vaccination. Interestingly, Bulgarelli et al. (Massimo Guidoboni lab), demonstrated that the ability of DC vaccine to increase the number of tumor infiltrating CD8^+^ T cells was impaired in metastatic melanoma patients refractory to immune-based therapies, including Ipilimumab (anti CTLA-4 antibody). On the other hand, despite a significant increase of intratumor CD8^+^ T cell density following DC vaccination, patients previously treated with chemotherapy or radiotherapy, failed to show a concurrent cytotoxic reactivation of T cells. The Authors discussed these results as partially explainable by DC-induced concomitant up-regulation of PD-L1 in cancer cells. Shinde et al. (Lalita Limaye lab) demonstrated that the use of anti-CTLA-4 monoclonal antibody in combination with DC vaccine strengthened the ability of DCs to successfully elicit tumor specific immune response in a multiple myeloma model. In this setting, autologous T cells showed signs of exhaustion, which can be rescued with CTLA-4 blockade. These studies clearly illustrate how the tumor immune microenvironment constantly shapes cancer cells and vice versa hence influencing the responsiveness to treatment. Therefore, the most critical challenge of immunotherapy now is the possibility to identify the specific mechanisms involved in the immune escape as well as critical targets responsible for local immunesuppression in each individual patient in order to define the most effective immunotherapeutic combination and personalize the treatment.

Castiello et al. (Cell Factory FaBioCell) thoroughly discussed the possibility to exploit the intratumor inoculation of antigen unloaded DCs, also called *in situ* DC vaccination, in combination with other anticancer therapies particularly with immunogenic pro-apoptotic agents. This strategy of vaccination takes advantage from the ability of inoculated DCs to uptake and process tumor associate antigens (TAAs) and neo-antigens directly released from tumor cells in the surrounding tumor microenvironment (TME). Intratumor injection of activated autologous DCs could efficiently enhance tumor cell elimination especially when used in combination with immunogenic cell death (ICD) inducing therapies (9) or other drugs able to improve cancer cells recognition and phagocytosis by DCs (10). It is well-known that ICD inducers, by promoting the tumor cell release of several molecules belonging to damage-associated molecular pattern (DAMPs), recruit DCs in the TME and improve their activation. In particular, in the present Research Topic, Lamberti et al. (Natalia B. Rumie Vittar lab) described for the first time the involvement of type I IFN pathway in the up-regulation of co-stimulatory signals and tumor-directed chemotaxis in DCs by melanoma cells following exposure to photodynamic therapy. Elucidating the intrinsic determinants of cancer cells undergoing ICD to enhance DC maturation and activation continues to keep the interest high with regard to the opportunity to exploit *ex vivo* ICD to improve DC-based vaccine efficacy.

Another therapeutically important aspect addressed in this special issue is the need to optimize the protocols for *ex vivo* monocyte-derived DC (mo-DC) generation in order to develop *ex vivo* a DC functional phenotype that is as close as possible to that of human DC subsets existing *in vivo*. On these grounds, Zeng et al. (Herbert Schwarz lab) comprehensively described the characteristics and discussed the potential clinical application of mo-DCs differentiated with the use of a CD137 ligand (CD137L) agonist. The authors highlighted the superior capacity of CD137L-DCs to stimulate T cell responses *in vitro* compared to conventional mo-DCs, obtained by exposure to granulocyte-macrophage colony-stimulating factor (GM-CSF) plus interleukin (IL)-4, and envisaged their potential therapeutic use as vaccine in combination with ICI immunotherapy.

The increased knowledge of molecular mechanisms, signaling pathways, epigenetic regulation and metabolic control of DC biology convincingly highlights the huge plasticity of these immune cells and indicates new modalities for *ex vivo* and/or *in vivo* DC manipulation in order to induce pro-inflammatory responses or revert tolerogenic/regulatory phenotypes.

In particular, tumor cells exploit different mechanisms and molecules to promote DC tolerization so as to evade immune surveillance, an aspect discussed in detail by DeVito et al. who gave a comprehensive overview about the important role of DC tolerization not only in tumor-mediated immune evasion, but also in generating immunotherapy resistance. According to these Authors, several lines of evidence highlight the pivotal role of DCs in the responsiveness to different immunotherapeutic approaches, indicating the need to extend ongoing efforts, which are mainly focused on manipulation of the effector phase of the antitumor immune response, also to the priming phase of the immune response by fully exploiting DC potential.

Notably, several molecular pathways involved in DC tolerization are also dysregulated in cancer cells being therefore targets of therapy. However, direct or indirect effects exerted by anticancer agents targeting these pathways on DCs still remain to be investigated. In this respect, Suryawanshi and Manicassamany thoroughly discussed the role of Wnt signaling cascade not only in regulating DC maturation, activation, and antigen presentation, but also in modulating the functions of other immune cells in TME. Interestingly, Fucikova et al. (Radek Spisek lab) debated the opportunity to take advantage of *ex vivo* DC tolerization to develop therapeutic vaccination against autoimmune diseases. In these cases, in fact, it is possible to exploit the ability of tolerogenic DC to stimulate regulatory T cell proliferation. These considerations suggest that a similar approach could be adoptable also in the treatment of lung chronic inflammatory diseases, such as chronic obstructive pulmonary disease or asthma in which an emerging role of DCs in maintaining unresolved inflammation was indicated (11).

All together, the contributions provided by this Research Topic of Frontiers in Immunology critically emphasized the strengths, but also highlighted relevant unresolved questions to be addressed to promote a broader application of DC-based vaccination in the clinical practice for cancer treatment. Moreover, the recent advances in our knowledge of DC tolerization described here clearly indicate DC-based vaccines may also be successfully used for the treatment of diseases characterized by chronic inflammation and dysregulated stimulation of immune responses as in autoimmunity. In conclusion, the central role of DCs in orchestrating immune responses continues to stimulate new strategies to exploit the functions of these immune cells for the development of more effective therapies for an increasing number of clinical settings.

## Author Contributions

All authors listed have made a substantial, direct and intellectual contribution to the work, and approved it for publication.

### Conflict of Interest

The authors declare that the research was conducted in the absence of any commercial or financial relationships that could be construed as a potential conflict of interest.
